# Evaluation of Vortioxetine on Global DNA Methylation in Maternal and Offspring Rats and In Silico Molecular Docking to Key Epigenetic Enzymes

**DOI:** 10.3390/ijms27020931

**Published:** 2026-01-17

**Authors:** Melih Günay, Merve M. Hız-Çelikliyurt, Gülsüm Akkuş, Şükrü Alperen Korkmaz

**Affiliations:** 1 Department of Molecular Biology and Genetics, Faculty of Science and Art, Istanbul Yeni Yüzyıl University, 34010 Istanbul, Türkiye; melih.gunay@yeniyuzyil.edu.tr; 2 Department of Medical Biology, Faculty of Medicine, Çanakkale Onsekiz Mart University, 17100 Çanakkale, Türkiye; mervemeliha@comu.edu.tr; 3 Department of Biology, Faculty of Science, Çanakkale Onsekiz Mart University, 17100 Çanakkale, Türkiye; 4 Department of Psychiatry, Faculty of Medicine, Çanakkale Onsekiz Mart University, 17100 Çanakkale, Türkiye

**Keywords:** toxicology, antidepressants, vortioxetine, DNA methylation, molecular docking, epigenetics, pregnancy, perinatal depression, maternal depression

## Abstract

Mothers face high depression risks during pregnancy, and untreated depression can harm both mother and baby. Vortioxetine is a novel antidepressant with a multimodal mechanism, unlike traditional ones. However, little is known about its safety and effectiveness in pregnancy due to limited preclinical and clinical data. This study investigated how maternal vortioxetine exposure during pregnancy affects DNA methylation in the brain tissue of mother and offspring rats. It also explored putative structural interactions of vortioxetine through molecular docking with key epigenetic enzymes to provide a hypothesis-generating context. Fifty female Sprague-Dawley rats were screened using a repeated forced-swim paradigm to characterize a passive stress-coping phenotype. They were then mated and randomly assigned to five groups (n = 10 each): vortioxetine at 0.5, 1.0, 2.0 mg/kg/day orally, saline control, and escitalopram (2.6 mg/kg/day orally) as a comparison. Treatments were given throughout pregnancy. On the day of cesarean section (G20), brain tissue was collected from both the mother and fetus. Global 5-mC levels were measured with ELISA (three replicates). The binding affinities and interaction motifs of vortioxetine and escitalopram with TET2, DNMT3A, and DNMT3B were analyzed via molecular docking. Global 5-mC levels in brain tissue did not differ between groups. However, a significant decrease in overall methylation was observed in offspring given the highest dose of vortioxetine (2.0 mg/kg/day). Docking analyses revealed that vortioxetine and escitalopram could bind strongly to TET2 and DNMT3A/3B; the observed reduction in global 5-mC was compatible with the hypothesis of altered de novo methylation pathways. The results show a specific dose threshold for the fetus. Low to moderate maternal exposures were not associated with detectable differences in global 5-mC under the current assay conditions, whereas high exposure was associated with hypomethylation in offspring. These findings underscore the importance of careful dose selection and mechanism validation for vortioxetine.

## 1. Introduction

Major depressive disorder (MDD) is a leading cause of disability among women worldwide, with its median age of onset often coinciding with reproductive (childbearing) years [1]. Depression occurring during pregnancy, commonly called perinatal depression, is one of the most frequent complications of pregnancy, with epidemiological studies indicating that about 8–12% of pregnant women meet the criteria for major depression [2,3]. Bennett et al. reported prevalence rates of 7.4% in the first trimester of pregnancy, 12.0–12.8% in the second trimester, and even higher rates in the first year after birth compared to the third trimester [4]. Zikic et al. found that 48.9% of pregnant women experienced depression, with 10.6% of cases occurring in the third trimester. The study revealed that the depression rate increased to 25% during the first month after birth, and 20.7% of depressed women experienced remission within six months [5]. However, the literature reports inconsistent rates of pregnancy-related depression. Okagbue et al.’s systematic review reports a range of 15–16.4%, whereas Gavin et al.’s meta-analysis indicates that major and minor depression across trimesters range from 6.5% to 12.9% [2,3]. Pregnancy is a particularly vulnerable time for mood disturbances; dramatic hormonal shifts, psychosocial stressors, and sleep disruptions may trigger depressive episodes, especially in women with a predisposition [6].

Untreated maternal depression can cause many adverse effects for both mother and child. Evidence indicates that untreated maternal depression can lead to poor birth outcomes, lower birth weight, decreased APGAR scores, growth delays, and developmental issues [7,8,9]. Recent meta-analyses confirm that antenatal depression nearly doubles the risk of preterm birth and small-for-gestational-age infants, emphasizing its significant impact on perinatal health [10]. Moreover, maternal depression has been linked not only to adverse obstetric outcomes but also to long-term neurodevelopmental risks in children, including emotional and cognitive challenges that may persist into adolescence [11,12]. These results highlight the importance of identifying and treating perinatal depression, as interventions can reduce both immediate obstetric risks and long-lasting effects on child development.

Effective management of antenatal depression is crucial for both maternal and fetal health. Selective serotonin reuptake inhibitors (SSRIs) are commonly prescribed as first-line antidepressants during pregnancy, used by approximately 6–8% of pregnant women [13]. While SSRIs are generally considered low-risk for teratogenicity, they have been associated with subtle neonatal adaptations, such as temporary withdrawal symptoms, and potential long-term neurobehavioral differences [14,15]. Modern antidepressants like vortioxetine, which may offer a more favorable profile, deserve careful consideration as treatment options during pregnancy. Early post-marketing data on vortioxetine’s use in human pregnancy, though limited, are promising. Animal studies have shown no teratogenic effects, and a case series of first-trimester exposures reported no congenital malformations [16]. Preliminary evidence indicates vortioxetine could be a viable alternative for perinatal depression, although larger controlled studies are needed to confirm its safety and efficacy.

The main perspective for studying the effects of antidepressants during the perinatal period is epigenetics. Epigenetic mechanisms, such as DNA methylation, histone modifications, and non-coding RNA regulation, show how environmental factors can influence gene expression without altering the DNA sequence [17,18]. During pregnancy, the fetal epigenome is particularly sensitive to maternal influences [19]. Maternal depression can produce high-stress hormones that reprogram fetal gene expression through epigenetic changes. Similarly, in utero exposure to antidepressants can cause lasting molecular modifications [20]. A potential mechanism linking SSRI treatment to fetal development outcomes involves alterations in DNA methylation patterns [19]. Evidence indicates that maternal mood disorders and treatments leave detectable epigenetic marks on offspring [20]. Studies have shown that prenatal exposure to maternal psychiatric illness or SSRIs can change DNA methylation in the placenta or cord blood, affecting genes involved in neurodevelopment and stress responses [20,21,22]. By analyzing epigenetic markers like DNA methylation, it becomes easier to understand how antidepressant treatments may either reduce or increase the molecular legacy of maternal depression.

To bridge the gap between clinical and molecular understanding, translational animal models of perinatal depression are crucial. Rodent studies allow controlled experiments on depression-like conditions during human pregnancy and therapeutic interventions [23]. In rodents, chronic stress paradigms or hormonal manipulations induce depression-like behaviors (such as lack of pleasure, anxiety, impaired maternal care) and associated neurobiological changes similar to those seen in depressed patients [24]. Notably, these stress models also exhibit epigenetic changes identical to those observed in human studies, including altered DNA methylation at stress-responsive genes (e.g., the glucocorticoid receptor and BDNF) [18]. These similarities support the use of animal data to infer human pathophysiology. Moreover, animal experiments help researchers identify causality. For example, by randomly assigning stress-exposed rodents (or rodents with a passive-coping phenotype) to receive or not receive antidepressants, scientists can determine how much of the offspring’s outcomes result from the mother’s mood versus drug exposure [17]. In epigenetics, rodent models are particularly valuable, as they enable detailed tissue analyses, such as examining fetal brain or placental DNA methylation, histone acetylation, or gene expression changes, to elucidate how maternal depression and its treatment influence developmental programming [25].

Vortioxetine is a new antidepressant with a multimodal mechanism of action that distinguishes it from traditional SSRIs. Pharmacologically, vortioxetine not only strongly inhibits the serotonin transporter, similar to SSRIs, but also directly modulates multiple serotonin (5-HT) receptor subtypes. Specifically, it acts as an agonist at 5-HT_1_A receptors and a partial agonist at 5-HT_1_B receptors, while antagonizing 5-HT_3_, 5-HT_7_, and 5-HT_1_D receptors [26]. Due to this unique profile, vortioxetine broadly enhances serotonergic neurotransmission across neural circuits, a mechanism believed to be a key driver of its clinical effects [27,28]. Beyond its impact on neurotransmitters, recent research highlights vortioxetine’s neuroplastic and neuroprotective capabilities. Preclinical studies indicate that it suppresses neuroinflammatory cytokines and reduces oxidative stress, suggesting an immunomodulatory role [29]. Some findings indicate that vortioxetine may induce molecular changes that enhance neuronal connectivity and resilience, potentially through epigenetic modifications such as demethylation of neurotrophic gene promoters or altered histone acetylation, which increases gene expression [30]. Given this profile, it is reasonable to consider that vortioxetine could also produce epigenetic effects as part of its therapeutic action. The elevation of BDNF and other plasticity-related genes induced by vortioxetine may involve epigenetic upregulation, a hypothesis that warrants further research [31].

In this study, we aimed to thoroughly investigate the effects of vortioxetine on behavioral and epigenetic outcomes under controlled conditions in rats, using a pre-mating repeated forced swim behavioral screening paradigm to characterize a passive stress-coping phenotype before gestational drug exposure. Therefore, our goal is to evaluate methylation levels caused by vortioxetine in both mother and offspring rats. Vortioxetine is believed to alter DNA methylation in the hippocampus, potentially affecting social behavior and the stress response in offspring. The insights gained can also improve our understanding of how epigenetic modifications interact with neuropharmacology to influence the development and treatment of depression across generations. Ultimately, this research can enhance our knowledge of the therapeutic potential and safety of vortioxetine during pregnancy, offering evidence-based guidance for antidepressant use in pregnant women.

## 2. Results

### 2.1. Forced Swim Test

**Figure 1:**
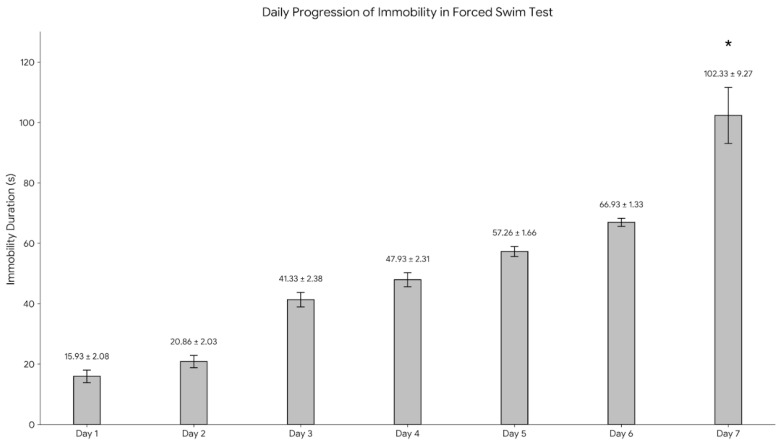
Daily progression of immobility duration (s) during the 7-day forced swim test protocol. Values are presented as Mean ± SD. While immobility gradually increased from Day 1 to Day 6, a statistically significant increase was observed on Day 7 relative to prior days (* *p* < 0.05).

The average duration of immobility (defined as cessation of active escape-directed behaviors, with only minimal movements to keep the head above water) during the 5-min forced swim test was recorded daily for seven consecutive days. This behavioral assessment was conducted on the entire drug-naïve cohort before randomization. For transparency, the baseline immobility data are presented stratified according to the groups to which animals were subsequently randomized (Groups 1–5), and Figure 1 summarizes the daily immobility times accordingly.

On the first day, the average immobility duration was 15.93 ± 2.08 s, which increased to 20.86 ± 2.03 s on the second day. On Day 3, it reached 41.33 ± 2.38 s; on Day 4, 47.93 ± 2.31 s; and by Day 5, 57.26 ± 1.66 s. The duration gradually increased to 66.93 ± 1.33 s on the sixth day, but these increases did not reach statistical significance. On the seventh day, the duration of immobility increased significantly to 102.33 ± 9.27 s.

### 2.2. Open Field Test

**Figure 2:**
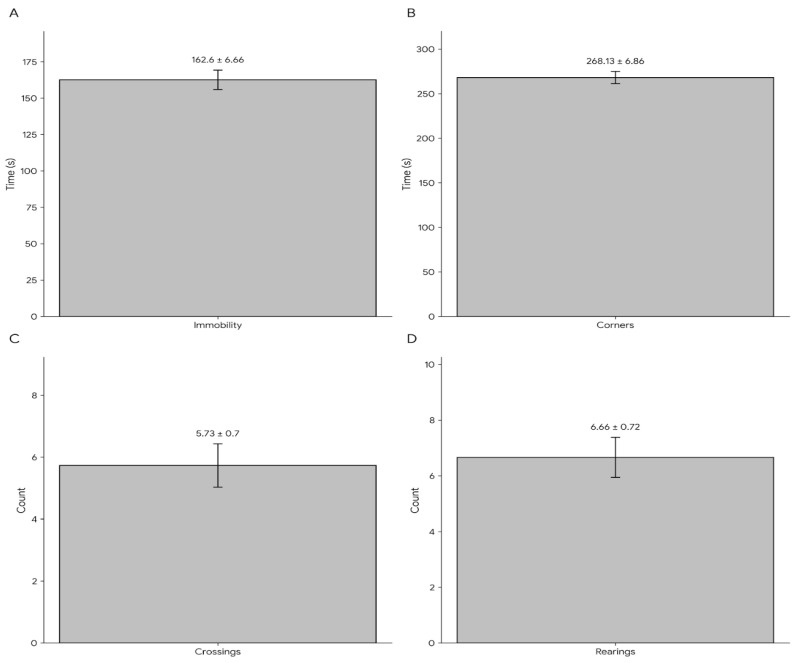
Open-field test outcomes were recorded during a 5-min session after completion of the repeated forced swim protocol and before mating/randomization. (**A**) Total immobility time (s); (**B**) time spent in corners (s); (**C**) number of squares crossed; (**D**) number of rearings. Bars represent mean ± SD (n = 50). No inferential statistics were performed across the different open-field endpoints; values are presented descriptively to characterize locomotor/exploratory readouts following the swim protocol.

Rats underwent open-field testing to assess locomotor activity/anxiety-like behavior. During the 5-min session, the number of line crossings, the times they stood on their hind limbs, and the time spent in the corners were recorded (Figure 2).

Because environmental exploration is directly linked to the frequency with which they stand on their hind limbs, we conclude that cognitive functions are intact. It is crucial to assess whether the animal in the open field wanders more around the edges or in the center. An animal removed from its familiar environment will avoid the middle area and move less. Our OFT results (greater time in corners/periphery and reduced center exploration) are more consistent with anxiety-like avoidance/thigmotaxis and provide a locomotor/emotionality context for interpreting FST immobility, rather than serving as a depression-specific confirmation [32,33].

In the open field test, the total inactivity time for the rats was 62.60 ± 6.66 s, while the time spent in the corners was 268.13 ± 6.86 s. The number of frame changes on all extremities of the animals was approximately 5.73 ± 0.70, and the number of rises on the hind extremities during the open field test was 6.66 ± 0.72 s.

### 2.3. Measuring 5-mC Global DNA Methylation

**Figure 3:**
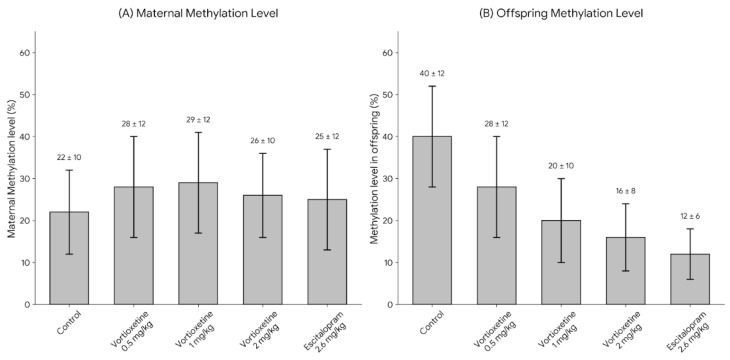
Effects of antidepressant treatment on global DNA methylation (5-mC) levels in hypothalamic tissue of dams and whole brain tissue of offspring. (**A**) Maternal methylation levels do not differ significantly between groups. (**B**) Offspring methylation levels significantly decreased in the Vortioxetine 20 mg and Escitalopram 20 mg groups compared to controls. Bars represent mean ± SD.

The experiment was conducted using the 5-mC DNA ELISA Kit (Zymo Research, Irvine, CA, USA; Cat. No. D5325). A standard curve was generated by mixing the negative (0% methylated) and positive (100% methylated) control DNA samples provided with the kit at different ratios. Absorbance readings were analyzed using GraphPad Prism (v9.0.2), and methylation levels were calculated from the standard curve. The methylation levels in maternal tissue samples were then measured (Figure 3). Although maternal methylation levels were not statistically significant, offspring global methylation levels showed a substantial reduction in the vortioxetine group (particularly at 2.0 mg/kg/day) compared with the other groups. Additionally, the results of vortioxetine were similar to those of escitalopram.

### 2.4. Molecular Docking Analysis Results

Molecular docking analyses of vortioxetine and escitalopram with TET2, DNMT3A, and DNMT3B were performed to generate a structural hypothesis for the observed in vivo global 5-mC pattern. The oxalylglycine ligand (PubMed CID: 3080614) was included as a reference ligand reported to inhibit TET enzymes, to benchmark the docking setup and pocket definition.

**Figure 4:**
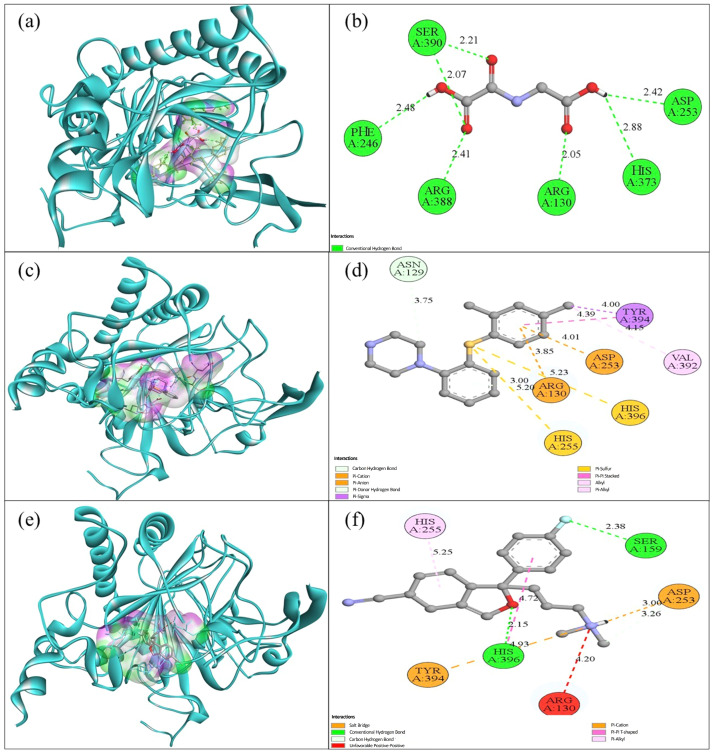
Molecular interaction between TET2 and oxalylglycine, vortioxetine, and escitalopram. (**a**) Three-dimensional (3D) binding pose of oxalylglycine within the TET2 catalytic pocket (TET2 shown as a cartoon; ligand shown as sticks; pocket surface displayed). (**b**) Two-dimensional (2D) interaction map of oxalylglycine, highlighting hydrogen-bond interactions with Ser390, Phe246, Arg388, Arg130, His373, and Asp253 (bond distances in Å are indicated). (**c**) 3D binding pose of vortioxetine within the TET2 pocket (protein cartoon; ligand sticks; pocket surface displayed). (**d**) 2D interaction map of vortioxetine, showing carbon–hydrogen bonds and π-mediated interactions (π–cation, π–anion, π–donor hydrogen bond, and π–sigma) primarily involving Asn129, Arg130, His255, Asp253, His396, Val392, and Tyr390 (distances in Å are indicated). (**e**) 3D binding pose of escitalopram within the TET2 pocket (protein cartoon; ligand sticks; pocket surface displayed). (**f**) 2D interaction map of escitalopram, indicating a salt bridge, conventional and carbon–hydrogen bonds with key residues including Ser159, His396, Asn253, Tyr394, and Arg130, as well as an unfavorable positive–positive contact with Arg130 (distances in Å are indicated).

Additionally, the S-adenosylhomocysteine (SAH) (PubChem CID: 439155) was included as a reference (a reaction product and reported DNMT inhibitor in vitro) to contextualize docking scores and interaction patterns. The predicted docking results indicated binding affinities of −6.019 kcal/mol for oxalylglycine, −6.980 kcal/mol for vortioxetine, and −7.459 kcal/mol for escitalopram. Oxalylglycine formed seven conventional hydrogen bonds, including with SER390 (2.21 Å), ARG130 (2.05 Å), ARG388 (2.41 Å), Phe246 (2.48 Å), ASP253 (2.42 Å), and HIS373 (2.88 Å). Meanwhile, vortioxetine interacted with ARG130 (3.00 and 5.23 Å), ASP253 (4.01 Å), TRY394 (4.00 and 4.39 Å), and HIS396 (3.85 and 5.20 Å) residues through pi-cation, pi-stacking, and different pi bonds, alkyl bonds, and hydrogen bonds (Figure 4).

When comparing docking poses/affinities with in vivo methylation findings, a potential interaction of vortioxetine/escitalopram with TET2 can be hypothesized. Docking suggested energetically favorable binding within the defined pocket; however, docking does not establish enzymatic inhibition. Therefore, the offspring hypomethylation pattern is reported as consistent with (but not proof of) a putative TET2-related mechanism, which requires biochemical validation.

**Figure 5:**
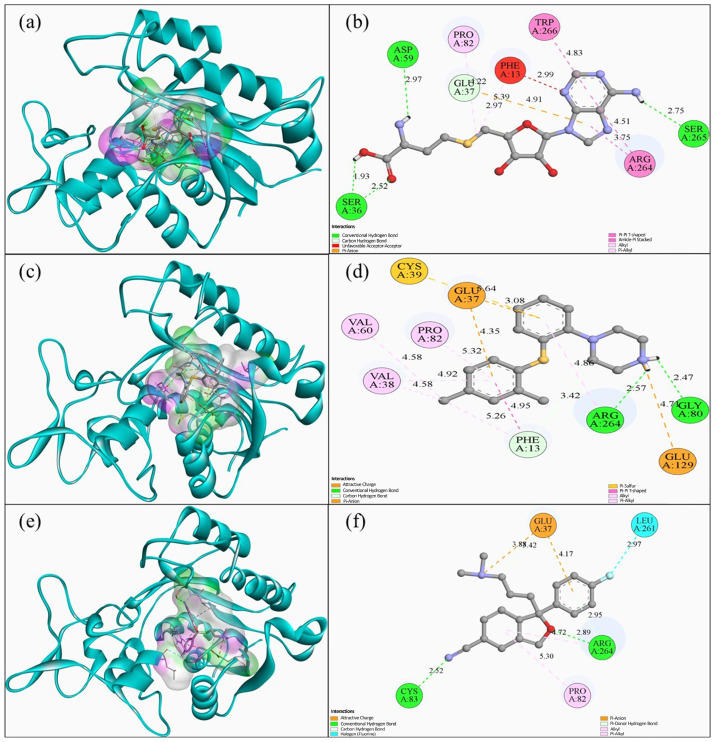
Molecular interaction between DNMT3A and S-adenosylhomocysteine, vortioxetine and escitalopram. (**a**) Three-dimensional (3D) binding pose of SAH in the DNMT3A catalyt-ic/SAM-binding pocket (DNMT3A shown as a cartoon; ligand shown as sticks; binding pocket surface displayed). (**b**) Two-dimensional (2D) interaction map of SAH, indicating hydrogen-bond and π/electrostatic contacts with Ser36, Asp59, Glu37, Pro82, Phe13, Arg264, Ser265, and Trp266 (interaction distances in Å are shown). (**c**) 3D binding pose of vortioxetine in the DNMT3A pocket (protein cartoon; ligand sticks; pocket surface displayed). (**d**) 2D interaction map of vortioxetine, showing key polar/electrostatic and π-mediated interactions involving Cys39, Glu37, Glu64, Val38, Val60, Pro82, Phe13, Arg264, Gly80, and Glu129 (distances in Å are shown). (**e**) 3D binding pose of escitalopram in the DNMT3A pocket (protein cartoon; ligand sticks; pocket surface displayed). (**f**) 2D interaction map of escitalopram, highlighting hydrogen-bond/electrostatic contacts and π-associated interactions with Cys39, Glu37, Glu64, Val38, Val60, Pro82, Phe13, Arg264, Gly80, and Glu129 (distances in Å are shown).

In this study, the binding affinities of DNMT3A for S-adenosylhomocysteine, vortioxetine, and escitalopram were calculated. S-adenosylhomocysteine was used as a positive control because it is a natural inhibitor of DNMT3A and DNMT3B [34]. The molecular docking analysis showed that SAH had the strongest binding affinity (−8.626 kcal/mol). Vortioxetine had a binding affinity of −7.844 kcal/mol, and escitalopram had a binding affinity of −8.222 kcal/mol. In evaluating the molecular interactions between the ligand and DNMT3A, SAH formed multiple conventional hydrogen bonds with residues such as SER36 (1.93–2.25 Å), ASP59 (2.97 Å), and SER265 (2.75 Å), as well as pi-anion and polar interactions. Additionally, vortioxetine interacted with residues such as ARG264 (3.42–4.86 Å), GLU37 (3.08–4.35 Å), and CYS39 via π-sulfur interactions and hydrogen bonds. Escitalopram formed interactions with GKY80 (2.47 Å), ARG264 (2.57 Å), and GLU129 (4.71 Å), including pi-stacked and alkyl interactions (Figure 5).

Subsequently, docking suggests that vortioxetine and escitalopram may bind to the DNMT3A/3B pockets under the chosen docking setup. Taken together, the docking results provide a hypothesis-generating mechanistic context consistent with the offspring hypomethylation pattern; however, in silico binding does not establish enzymatic inhibition or causality in vivo. Together, the docking and methylation results are qualitatively consistent with a testable mechanistic hypothesis, but do not establish correlation or causality. Although there was no significant difference in global methylation levels between maternal and offspring rats, offspring showed a dose-dependent effect on methylation. This hypomethylation in offspring is consistent with docking results, in which escitalopram showed binding affinity to DNMT3A comparable to that of SAH and formed multiple stabilizing interactions with key catalytic-site residues.

**Figure 6:**
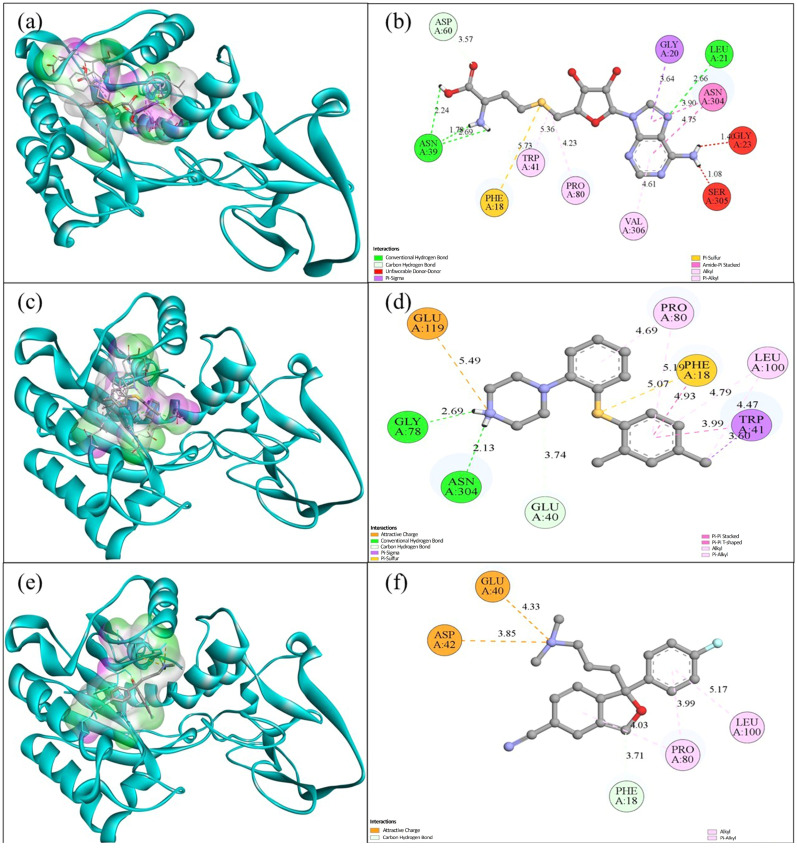
Molecular interaction between DNMT3B and S-adenosylhomocysteine, vortioxetine, and escitalopram. (**a**) Three-dimensional (3D) binding pose of SAH in the DNMT3B catalyt-ic/SAM-binding pocket (DNMT3B shown as a cartoon; ligand shown as sticks; binding pocket surface displayed). (**b**) Two-dimensional (2D) interaction map of SAH, showing convention-al/carbon hydrogen bonds with Asn39 and contacts involving Asp60, Gly20, Leu21, Asn304, Gly23, and Ser305, as well as π/hydrophobic interactions with Phe18, Trp41, Pro80, and Val306; unfavorable donor–donor contacts are indicated where observed (interaction distances in Å are shown). (**c**) 3D binding pose of vortioxetine in the DNMT3B pocket (protein cartoon; ligand sticks; pocket surface displayed). (**d**) 2D interaction map of vortioxetine, highlighting polar/electrostatic interactions (including an attractive charge contact with Glu119 and hydrogen-bonding with Gly78 and Asn304) and π/hydrophobic interactions primarily involving Trp41, Phe18, Pro80, and Leu100 (distances in Å are shown). (**e**) 3D binding pose of escitalopram in the DNMT3B pocket (protein cartoon; ligand sticks; pocket surface displayed). (**f**) 2D interaction map of escitalopram, indicating electrostatic/attractive charge interactions with Glu40 and Asp42 and additional hydropho-bic/π-associated contacts with Pro80 and Leu100, with Phe18 positioned proximal to the binding region (distances in Å are shown).

The binding affinities and interactions of DNMT3B with SAH, as well as those of vortioxetine with escitalopram, were analyzed. Their binding affinities were −8.340 kcal/mol, −9.601 kcal/mol, and −8.984 kcal/mol, respectively. The analysis of molecular interactions between SAH and DNMT3B revealed various stabilizing interactions, including conventional hydrogen bonds with ASN39 (1.78–2.69 Å) and ASP60 (3.57 Å), as well as pi-stacked and pi-sigma interactions with aromatic residues such as TRP41 and VAL306. Vortioxetine showed a stronger binding affinity than SAH and formed extensive interactions with residues like GLY78 (2.69 Å), ASN304 (2.13 Å), and GLU119 (5.49 Å), along with multiple pi-sigma, pi-sulfur, pi-pi stacked, pi-pi T-Shaped, and alkyl bonds, adopting an interaction-rich pose compatible with in silico pocket engagement within the modeled DNMT3B binding site under the chosen docking setup. Similarly, escitalopram also exhibited a slightly higher binding affinity than SAH and interacted with residues such as GLU40 (4.33 Å), ASP42 (3.85 Å), and PHE18 (3.18 Å), as well as several hydrophobic contacts with LEU100 and PRO80 (Figure 6).

These findings suggest that both vortioxetine and escitalopram have higher affinity and more diverse interactions with DNMT3B than SAH. The numerically favorable docking score and interaction pattern of vortioxetine are consistent with in silico engagement of the DNMT3B pocket; however, docking alone cannot infer inhibitory potency or functional modulation of DNMT3B-mediated methylation, which requires biochemical and/or cellular validation. Molecular docking results provide a structural, hypothesis-generating rationale to prioritize DNMT3A/3B (and TET2) for follow-up testing in relation to the offspring hypomethylation pattern; direct enzyme modulation and functional epigenetic effects remain to be demonstrated experimentally.

## 3. Discussion

Given the significant increase in antidepressant use, it is crucial to understand how exposure to antidepressants during pregnancy affects the fetus to better inform treatment decisions for women experiencing depression at this time. Consistent with our primary finding, vortioxetine did not alter global 5-mC levels in the maternal brain. In contrast, offspring exposed to 2.0 mg/kg/day (approximately 20 mg/day in humans) showed a notable reduction in global methylation (measured by ELISA in brain tissue). In our rat model, this offspring-specific, dose-dependent pattern suggests fetal-window sensitivity and a potential dose threshold. Building on these findings, we further examined the mechanistic basis by molecularly docking methylation regulators (TET2, DNMT3A/3B) to contextualize the observed effects in offspring.

Although no study is directly comparable to our findings, the effects of different antidepressants on gene-specific methylation levels have been examined, particularly in perinatal cohorts. Critically, most human data are locus-specific and derived from peripheral tissues (cord blood/placenta), whereas our assay quantifies global 5-mC in the brain; accordingly, our results should be interpreted as complementary rather than directly comparable to these locus-specific signals. This methodological heterogeneity constrains direct comparison of effect sizes while clarifying why different tissues/assays can yield divergent readouts. For instance, Galbally et al. [35] reported reduced placental OXTR-promoter methylation following antidepressant exposure, and Soubry et al. [36] linked differences in IGF2 methylation to dysregulated fetal growth signaling. Consistent with a targeted, gene-level signal, methylation changes were observed at CpG sites in the TNFRSF21 gene, which is involved in apoptosis and the development of neurons and lymphocytes; in antidepressant-exposed neonates, methylation levels at these CpG sites decreased by 1.9%, while a 3% increase was observed in another CpG site.

Region of CHRNA2 [37]. At the epigenome-wide level, large cohorts report subtle neonatal differences associated with maternal mood/antidepressants, with directions and magnitudes varying by tissue and analytic pipeline, and some signals attenuating after multiple-testing correction (e.g., Cardenas et al. [38]). Taken together, this heterogeneous literature aligns with our pattern—unchanged maternal global methylation but offspring-specific reductions at the highest vortioxetine exposure—supporting a model in which tissue, timing, and dose dictate whether perinatal effects appear as locus-specific marks or broader global 5-mC shifts.

Molecular docking analyses in this study suggest that vortioxetine and escitalopram can adopt energetically favorable binding poses within the selected pockets of TET2, DNMT3A, and DNMT3B, providing a structural, hypothesis-generating context for the observed global 5-mC pattern. TET2 is a key enzyme in active DNA demethylation, catalyzing the oxidation of 5-methylcytosine to 5-hydroxymethylcytosine [39]. Under our docking setup, vortioxetine (−6.980 kcal/mol) and escitalopram (−7.459 kcal/mol) yielded docking scores comparable to, and numerically lower than, oxalylglycine (−6.019 kcal/mol), which has been reported as a TET2 inhibitor [40]. Importantly, docking scores and poses alone do not establish binding kinetics or functional outcomes (e.g., inhibition or activation), nor do they demonstrate causal linkage to in vivo methylation changes. Therefore, rather than concluding that TET2 inhibition is sufficient to account for the observed offspring hypomethylation, our findings support a testable working hypothesis that direct interactions with TET2 may be among several plausible pathways contributing to this phenotype in vivo. This interpretation is consistent with prior work highlighting that perturbation of TET2 activity can influence DNA methylation/hydroxymethylation balance [41]. DNMT3A and DNMT3B are essential enzymes responsible for de novo DNA methylation during development [42]. Docking similarly indicated favorable poses and scores for vortioxetine and escitalopram within DNMT3A and DNMT3B, compared with S-adenosylhomocysteine, with interaction patterns (e.g., hydrogen bonding and π-stacking) compatible with stable occupancy at the modeled sites [43]. These in silico observations are compatible with—yet do not prove—a model in which gestational exposure could influence de novo methylation pathways in the embryo. The minimal change in global methylation in maternal tissue, in contrast to hypomethylation in offspring, suggests developmental-stage-specific sensitivity and/or maternal–placental–fetal compartmentalization of exposure. However, confirming placental transfer and any functional modulation of DNMT/TET enzymes will require dedicated in vivo and biochemical validation (e.g., placental/fetal drug quantification, DNMT/TET activity assays, and measurements of 5-hmC alongside locus-specific methylation profiling). Collectively, our results align with the broader concept that antidepressant exposure during pregnancy may be associated with epigenetic alterations with potential developmental relevance [44], while underscoring that mechanistic inference remains provisional in the absence of direct functional assays.

Mechanistically, the offspring-specific hypomethylation may arise through multiple, non-mutually exclusive pathways. First, antidepressant exposure could indirectly influence the fetal epigenome via maternal–placental–fetal signaling (e.g., stress hormones, inflammatory/redox pathways) that modulate the activity or expression of the DNA methylation/demethylation machinery. Second, and more speculatively, intercellular communication via extracellular vesicles (EVs) has been proposed as a route for epigenomic reprogramming: EV cargo (RNAs/proteins/DNA) can remodel recipient-cell epigenetic profiles, including DNA methylation, and EV-associated components linked to DNMT/TET regulation have been reported. While our study did not measure EVs, future work could quantify placental and circulating EVs and characterize their epigenetic cargo in parallel with locus-specific methylation assays to test this hypothesis [45,46,47].

Vortioxetine’s pharmacokinetics, marked by a long half-life and extensive liver metabolism via cytochrome P450 enzymes (CYP2D6 and CYP3A4), suggest the potential for prolonged maternal–fetal exposure. While the accumulation of the parent drug and/or its metabolites in fetal compartments is a plausible contributor to effects on the offspring, this remains a hypothesis that requires direct measurement. Individual differences in maternal metabolism, placental transfer efficiency, and fetal clearance are additional factors that could influence methylation readouts. Therefore, a pharmacokinetic bridge is necessary: to measure vortioxetine and key metabolites in maternal plasma, placenta, cord blood, and fetal brain at delivery; to connect exposure metrics (Cmax, AUC, placenta–fetus ratios) with 5-mC/5-hmC changes; and to use population pharmacokinetic/ pharmacodynamic (PK/PD) modeling to test exposure–response thresholds. Importantly, this study did not measure PK parameters, which limits the ability to link dose causally to epigenetic outcomes; future research should include these PK assessments prospectively to determine whether the observed offspring hypomethylation at the highest dose is due to threshold exposure, timing of exposure, or developmental vulnerability.

This study has several limitations that should be acknowledged. First, the sample sizes in each experimental group were small, which may limit the generalizability of the findings. Second, locomotor activity was assessed only as a post-screening open-field readout; we did not collect both pre- and post-screening locomotor measures. Although behavioral test order and repeated exposure can influence subsequent forced-swim behavior, future studies could incorporate a dedicated repeated locomotor assessment to further disentangle stress-induced behavioral changes from subtle motor confounds. Third, our study examined only overall DNA methylation levels, without analyzing specific gene regions or pathways, which could provide more detailed insights into the epigenetic effects of vortioxetine. Fourth, we did not investigate long-term behavioral or developmental outcomes in offspring, which are essential for understanding the broader implications of prenatal vortioxetine exposure. Fifth, the pharmacokinetic parameters of vortioxetine, including maternal blood levels, placental transfer, and fetal tissue distribution, were not measured. A limitation is that we quantified global DNA methylation (5-mC) but did not perform gene-expression profiling (e.g., RT-qPCR or transcriptomics) or locus-specific methylation assays. Future studies should integrate targeted RT-qPCR (e.g., Bdnf, Dnmt3a/3b, Tet2) and gene- and locus-specific methylation analyses to link global 5-mC changes with functional transcriptional outcomes. Finally, the potential influence of genetic differences in drug metabolism on methylation outcomes was not examined; such differences could account for interindividual variation in vortioxetine response. Addressing these limitations in future research will enhance the understanding of the safety and effectiveness of vortioxetine during pregnancy.

Importantly, the forced swim test primarily indexes stress-coping strategy rather than a direct analog of human depression. Increased immobility can reflect an energy-conserving passive coping style and has been discussed in the context of learned helplessness/learned passivity, particularly under repeated exposure paradigms, rather than unequivocally “depression-like” behavior. Therefore, in the present study, we used the FST as a standardized behavioral screen to establish a stress-related passive-coping phenotype before mating and pharmacological exposure, and we interpret downstream biological findings within this translational context [48,49].

Besides the limitations of this study, it also possesses notable strengths. Firstly, the incremental-dose design with clinical relevance (5/10/20 mg/kg vortioxetine and 20 mg/kg escitalopram as the active comparator) facilitated interpretation of epigenetic outcomes in the context of dose–response relationships. Secondly, concurrent sampling of maternal and fetal brain tissues on the 20th day of gestation enabled direct examination of maternal-fetal separation. Thirdly, measuring global 5-mC in triplicate using ELISA enhanced technical reliability. Fourthly, triangulating wet lab results with docking analyses targeting TET2/DNMT3A-3B strengthened the biological plausibility framework. Lastly, the two-stage behavioral validation (FST/OFT) supported the translational interpretation by confirming the pre-pregnancy passive stress coping phenotype (FST immobility as a coping style readout). Collectively, these elements improve the biological consistency of the pattern “global methylation preservation in mothers but decreased exposure in the fetus.” From a clinical perspective, they imply the “lowest effective dose” strategy during pregnancy and practical approaches such as epigenetic monitoring (e.g., cord blood 5-mC/5-hmC, placental profile), at birth or early childhood.

## 4. Materials and Methods

### 4.1. Animals

Adult female Sprague-Dawley rats (10 weeks old at study onset, weighing 150–200 g) were used in this study. We selected 10-week-old female Sprague-Dawley rats because this age corresponds to young adulthood in rats (post-adolescent, sexually mature), thereby minimizing confounding effects of ongoing adolescent neurodevelopment while avoiding age-related reproductive changes that may independently influence epigenetic profiles [50]. Additionally, this age was intentionally selected because vortioxetine and escitalopram primarily exert their effects via serotonergic mechanisms; adolescence is associated with substantial maturation-related changes in serotonin transporter binding and 5-HT receptor signaling, which can introduce age-dependent variability in responses to serotonergic drugs. Therefore, restricting the experiment to post-adolescent animals improves the interpretability of drug effects on a comparatively mature serotonergic system. In addition, females at this age are typically sexually mature, supporting reliable mating and pregnancy. All animals had free access to water and food. They were obtained from the ÇOMÜ Experimental Research Application and Research Center in Çanakkale, Türkiye. The Ethical Committee for the Experimental Animals at Çanakkale Onsekiz Mart University approved the protocol (approval number: 2018/04, dated 13 April 2018).

### 4.2. Stress-Coping Behavioral Assessment Before Mating

#### 4.2.1. Forced Swim Test (FST)

The forced swim test (FST) is widely used to quantify stress-coping strategies and antidepressant-like behavioral effects in rodents. Because the interpretation of immobility as a direct model of human depression is debated, we used the FST as a standardized behavioral assay to establish a stress-related depressive-like coping phenotype before mating and pharmacological exposure. All procedures were conducted with welfare refinements and in line with current reporting standards [51,52].

After a 7-day acclimation period, animals underwent a repeated forced-swim protocol consisting of a 15-min pre-exposure session (FST day 0) followed by seven consecutive daily 5-min sessions (FST days 1–7). On day 0, each rat was individually placed in a container (60 cm × 60 cm × 60 cm) filled with water at 24 ± 1 °C for 15 min.

They were then dried and returned to their cages. After a 24-h rest period, the test was repeated under the same conditions for 5 min daily for seven consecutive days (days 1–7). Active behaviors (swimming and climbing) and passive behavior (immobility) were scored daily and analyzed. Immobility was operationally defined as the absence of active escape-directed behaviors (e.g., swimming or climbing), with the animal making only the minimal movements necessary to keep its head above the water. Given the ongoing debate, immobility was interpreted here as a shift toward passive stress-coping/energy conservation rather than a direct proxy for depression severity [53]. Stress-related depressive-like coping phenotype was considered established based on the expected pattern of increased immobility (passive coping) across repeated sessions, as commonly interpreted in the FST literature. The OFT was subsequently used as a locomotor/exploratory readout to help exclude gross motor impairment as a confound when interpreting FST behavior [51,54].

#### 4.2.2. Open Field Test (OFT)

**Figure 7:**
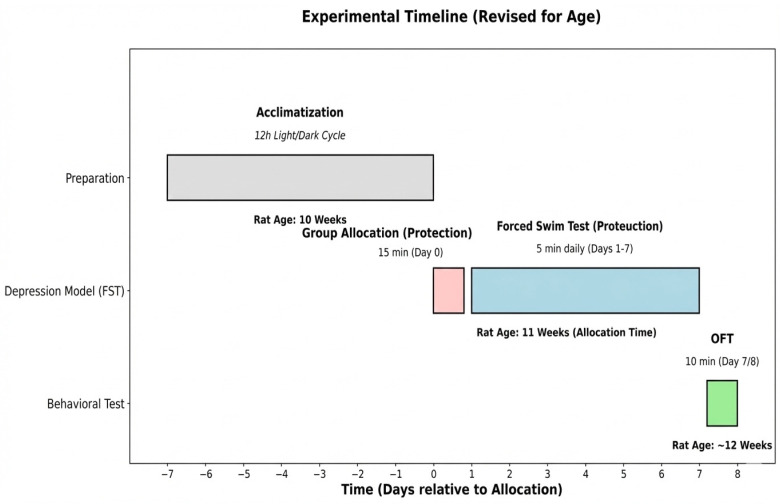
Experimental Timeline.

Following the FST, the Open Field Test (OFT) was performed on all experimental groups. Each rat was placed individually in the corner of a square Plexiglas box (60 cm × 60 cm × 60 cm) in a well-lit room and allowed to explore the open field arena for 10 min freely. Locomotor activity parameters were computed across the entire session. In contrast, anxiety-related indices (e.g., time in center/center entries) were primarily evaluated within the first 5 min, when novelty-driven emotionality is most prominent. The time spent in the central area of the box (12.5 cm × 12.5 cm) and the distance traveled during the test were recorded and analyzed using a video camera. During the first five minutes, the following parameters were measured: the number of hind limb elevations, the number of line crossings, and the time spent in the corners. The OFT was included as a locomotor control to interpret forced-swim behavior in the absence of drug-related motor effects (all animals were drug-naïve during the behavioral screening phase). We did not conduct an additional pre-FST open-field session to avoid potential order/carry-over effects across behavioral assays and to minimize additional novelty/stress exposure immediately before mating [55,56]. The experimental timeline for rats is schematically shown in Figure 7.

### 4.3. Randomizing Study Groups

After completion of the forced swimming test (FST) and the open field test (OFT), the 50 female rats (11-week-old) were randomized into five groups (n = 10 per group) before mating. Drug administration was initiated only after pregnancy confirmation (defined as gestational day 0, GD0) and continued once daily until GD20.

The study groups are as follows:

- Group 1 (n = 10): Vehicle control (0.9% Saline),
- Group 2 (n = 10): Vortioxetine 0.5 mg/kg/day (≈5 mg/day human equivalent)
- Group 3 (n = 10): Vortioxetine 1.0 mg/kg/day, (≈10 mg/day human equivalent)
- Group 4 (n = 10): Vortioxetine 2.0 mg/kg/day, (roughly ≈ 20 mg/day human equivalent)
- Group 5 (n = 10): Positive control (escitalopram oxalate 2.6 mg/kg/day; ≈20 mg/day human equivalent)

Vortioxetine hydrobromide and escitalopram oxalate were obtained from a community pharmacy. The drugs were administered orally by gavage after being dissolved in saline (0.9% NaCl). Vortioxetine was administered at 0.5, 1.0, and 2.0 mg/kg/day, whereas escitalopram was administered at 2.6 mg/kg/day as the positive control. Group 1 received the same volume of saline.

### 4.4. Pregnancy of Rats

After pre-mating behavioral screening using the repeated FST (passive stress-coping phenotype) and the subsequent OFT assessment, spontaneous mating was initiated by placing one male rat in each cage across all experimental groups. Pregnancy was monitored daily through vaginal smears, and pregnant animals were identified and separated into their respective groups. No pharmacological treatment was administered before mating; treatments were initiated only after pregnancy confirmation. Following pregnancy confirmation (the day spermatozoa were detected in the vaginal smear was defined as gestational day 0, GD0), animals received once-daily oral gavage of vortioxetine (Groups 2–4), escitalopram (Group 5), or saline (Group 1), starting on GD0 (i.e., the first day of pregnancy) and continuing until GD20. Accordingly, all animals were drug-naïve throughout behavioral induction/screening and mating, and only received vortioxetine, escitalopram, or saline after pregnancy confirmation (GD0–GD20). Ongoing pregnancies were monitored through abdominal palpation on days 10–12 and by observing mammary gland development by day 14. Since the gestation period is 21 days, pregnancies were terminated via cesarean section on GD20.

### 4.5. Tissue Collection

Maternal and fetal brain tissue samples were collected immediately following cesarean sections on gestational day 20. In maternal rats, the hippocampus was microdissected as the target region because of its established role in depression and epigenetic regulation. In newborn offspring (P0–P1), whole-brain homogenates were used because hippocampal boundaries could not be reliably distinguished stereotaxically, and the small tissue volume necessitated consistent sampling to ensure reproducibility. A standardized workflow for DNA extraction and methylation measurement was applied to all samples to minimize technical variation and loss of sections. Methylation measurements in offspring reflect global brain methylation levels rather than region-specific signals. The tissue samples were placed into 2 mL Eppendorf tubes (Eppendorf SE, Hamburg, Germany), snap-frozen in liquid nitrogen, and stored at −80 °C until further analysis.

### 4.6. DNA Isolation

DNA was extracted from the tissue using the QIAamp DNA Extraction Kit (Qiagen, Cat: 56304, Hilden, Germany) according to the manufacturer’s protocol. The concentration and purity of the isolated DNA were determined using a MultiskanGO spectrophotometer (Thermo Fisher, Waltham, MA, USA). The 260/280 and 260/230 ratios were used to assess DNA quality. The 260/280 ratio indicates protein contamination, while the 260/230 ratio suggests potential organic contamination. For optimal DNA purity, the 260/280 ratio ranged from 1.8 to 2, and the 260/230 ratio was approximately 2.

### 4.7. Global 5-Methylcytosine DNA Analysis

The total level of 5-mC was measured using the enzyme-linked immunosorbent assay (ELISA) method with the 5-methylcytosine DNA ELISA Kit (Cat: D5325, Zymo Research, Orange, CA, USA) according to the manufacturer’s protocol. DNA concentrations were determined by measuring absorbance at 260 nm and converting the readings to ng/μL using a factor of 50. The samples were then diluted with sterile deionized water to a final concentration of 20 ng/μL for assessing 5-mC methylation levels. Each tissue measurement was performed three times.

### 4.8. Molecular Docking Analysis

Molecular docking analysis of three target proteins, TET2 (PDB ID: 5d9y), DNMT3A (PDB ID: 6f57), and DNMT3B (PDB ID: 5ciy), was retrieved from the RCSB Protein Database (PDB). Proteins were prepared for docking studies using the DockPrep tool in ChimeraX v1.9. All water molecules, ions, and ligands were removed to prevent interference during the docking analysis. In addition, polar hydrogen atoms were added to the receptor proteins. Additionally, the 3D structures of the ligands used in docking analysis, which are vortioxetine (PubChem CID: 9966051), escitalopram (PubChem CID: 146570), the TET2 inhibitor oxalylglycine (PubChem CID: 3080614), and the DNMT inhibitor S-adenosylhomocysteine (PubChem CID: 439155), were downloaded from the PubChem chemical database. Before docking studies, all ligand structures were geometrically optimized using Avogadro v2.0, and ligands were prepared for docking analysis with the DockPrep tool in ChimeraX v1.9 and saved in .mol2 format. After preparation, molecular docking was performed using AutoDock Vina (v1.2.5) in the SwissDock online docking tool (Swiss Institute of Bioinformatics). For the docking analysis of TET2-Oxalylglycine, TET2-Vortioxetine, and TET2-Escitalopram, the docking grid was defined to encompass the protein’s active site and binding pocket. The grid parameters were set to a box size of X = 30, Y = 30, and Z = 30, centered at X = −18.0, Y = 4.0, and Z = −37.0, with a sampling exhaustiveness value of 24. The grid box dimensions (30 × 30 × 30 Å) were selected to fully cover the catalytic pocket and adjacent subpockets while providing sufficient margin for ligand translation/rotation, thereby minimizing boundary artifacts and avoiding artificial restriction of potential binding poses. Exhaustiveness was set to 24 as a pragmatic compromise between computational cost and thorough conformational sampling, and the same exhaustiveness value was applied across all targets to ensure methodological comparability. For the docking simulation of DNMT3A with S-adenosylhomocysteine, vortioxetine, and escitalopram, the grid was configured to target the protein’s active site and binding pocket specifically. The grid dimensions were set to a box size X = 30, Y = 30, and Z = 30, centered at X = −549.0, Y = 24.0, and Z = 171.0. A sampling exhaustiveness value of 24 was applied to achieve an optimal balance between computational efficiency and thorough conformational search. Additionally, for the docking analysis of DNMT3B with S-adenosylhomocysteine, vortioxetine, and escitalopram, the grid was designed to cover the protein’s active site and binding pocket. The grip dimensions were set to a box size X = 30, Y = 30, and Z = 30, centered at X = 24.0, Y = −6.0, and Z = 337.0. A sampling exhaustiveness value of 24 was employed to ensure adequate conformational sampling while maintaining computational efficiency. Binding affinities and RMSD values were calculated, and the docking results were visualized using BIOVIA Discovery Studio Visualizer v24.1.0.23298. The protein-ligand interaction was determined, and the interatomic distance was computed using BIOVIA Discovery Studio Visualizer.

### 4.9. Statistical Analyses

Statistical analyses were performed using IBM SPSS v24.0 (IBM Corp., Armonk, NY, USA). Data are reported as mean ± SD. Distributional assumptions were assessed using visual inspection and the Shapiro–Wilk test. For the repeated FST sessions (seven consecutive days), changes in immobility over time were evaluated using repeated-measures ANOVA (within-subjects factor: day), with a Greenhouse–Geisser correction applied when sphericity was violated, followed by Bonferroni-adjusted post hoc comparisons. Between-group comparisons for maternal and offspring molecular outcomes (e.g., ELISA-derived global 5-mC levels) were conducted using one-way ANOVA with Tukey’s multiple-comparisons test (or Kruskal–Wallis with Dunn’s post hoc test when assumptions were not met). All tests were two-tailed, and *p* < 0.05 was considered statistically significant.

## 5. Conclusions

Our findings demonstrate that vortioxetine does not alter maternal global 5-mC relative to global DNA methylation in pregnant rats, but shows a significant, dose-dependent effect on offspring methylation at higher doses. These results suggest that vortioxetine may be safely used at lower doses during pregnancy; however, higher doses require caution due to their potential to induce epigenetic alterations in offspring. Our findings underscore the crucial need for precise dose regulation and encourage further studies to investigate the pharmacokinetics and long-term effects of vortioxetine during pregnancy. This study expands our understanding of the epigenetic effects of antidepressant use during pregnancy and provides a foundation for refining treatment strategies for maternal depression.

Taken together, these findings support cautious dose selection when considering vortioxetine exposure in pregnancy models: lower exposures appeared epigenetically neutral in the dams, whereas higher exposures were associated with hypomethylation in the offspring. Because pharmacokinetic parameters and locus-resolved methylome data were not collected, definitive causal inferences cannot be drawn. Our data should be interpreted as a proof of concept and a hypothesis-generating study, demonstrating that gestational exposure to vortioxetine and escitalopram is associated with measurable changes in global DNA methylation in offspring tissues. Potential downstream transcriptional consequences remain to be determined in future gene-expression studies, rather than establishing a definitive causal molecular mechanism. Future studies should integrate maternal–placental–fetal pharmacokinetics, concurrent assays of 5-mC/5-hmC and DNMT/TET activities, gene-resolved methylome/transcriptome profiling, and longitudinal neurobehavioral follow-up to determine whether the observed global methylation change translates into persistent functional outcomes. These data expand the evidence base on antidepressant-associated epigenetic modulation during pregnancy and provide a framework for refining exposure levels in translational research.

## Data Availability

The data supporting the findings of this study are available from the corresponding author upon reasonable request.
